# Hypophosphatemic Rickets: Presenting Features of Fanconi—Bickel Syndrome

**DOI:** 10.1155/2011/314696

**Published:** 2011-10-18

**Authors:** Mahua Roy, K. Bose, D. K. Paul, Puja Anand

**Affiliations:** Department of Pediatric Medicine, Dr. B. C. Roy Postgraduate, Institute of Pediatric Sciences, West Bengal, Kolkata 700054, India

## Abstract

Fanconi-Bickel Syndrome (FBS) is a rare variety of glycogen storage disease (GSD). Characterized by massive hepatomegaly due to glycogen accumulation, severe hypophosphatemic rickets, and marked growth retardation due to proximal renal tubular dysfunction. We report a young boy presented as hypophosphatemic rickets with hepatomegaly and subsequently diagnosed as FBS.

## 1. Introduction

FBS is a rare form of GSD (Type-XI) [1, 2]. It is known since 1940 as hepatorenal glycogenesis with proximal renal tubular dysfunction [3, 4]. The pathogenic mutation of GLUT 2 gene of hepatocytes, beta cells of pancreas and renal tubules were discovered in 1997. We report a young boy who is presented with clinical features of rickets along with hepatomegaly, growth retardation, and developmental delay. Investigations detected rachitic changes with calciuria, phosphaturia, glycosuria, mild metabolic acidosis, and liver biopsy features were highly suggestive of GSD.

## 2. Case Report

An 18-month-old boy was admitted for evaluation of resistant rickets. The boy had been treated in a local hospital with 2 mega doses of vitamin D without any clinical or radiological improvement. Detailed history taking revealed that he had failure to thrive, abdominal distension, and motor developmental delay. The baby was the first issue of nonconsanguineous Hindu parents and had been born through normal vaginal delivery at term (37 weeks) with birth weight of 2.5 kg; without any significant antenatal and postnatal history. According to the parents, exclusively breastfeeding was continued for only 2 months followed by top feeding. Family diet was started from 12 months of age. Social smile had developed at 3 months, head holding at 6 months, and sitting with support at 11 months. He could not sit without support till the age of 18 months.

**Figure 1:**
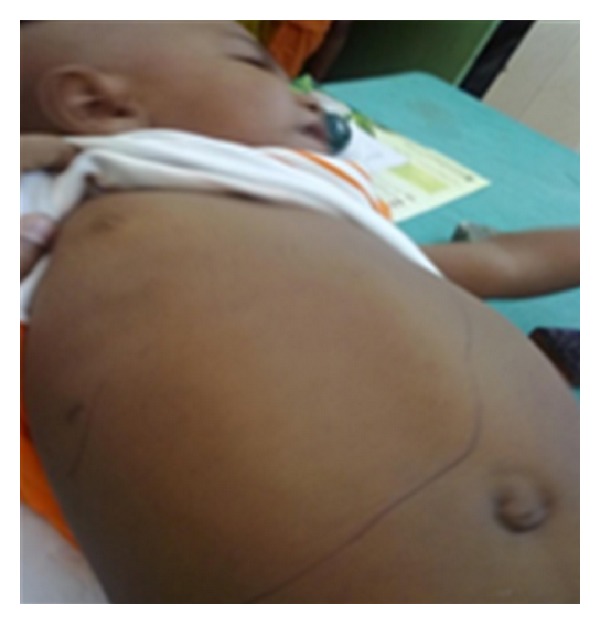
Showing child with huge hepatomegaly.

Clinical examination detected; weight 8 kg, head circumference 42 cm, and length 72 cm (all were below and 3rd percentile). Anterior fontanel was wide open, widening of wrist and ankle joints with genu valgum was noticed. Abdominal examination detected hepatomegaly (10 cm) which was firm, smooth, and nontender; without any other organomegaly or ascites (Figure 1). Nervous system examination showed generalized hypotonia. The developmental age of the child was approximately seven months. Other systems revealed no abnormality.

**Figure 2:**
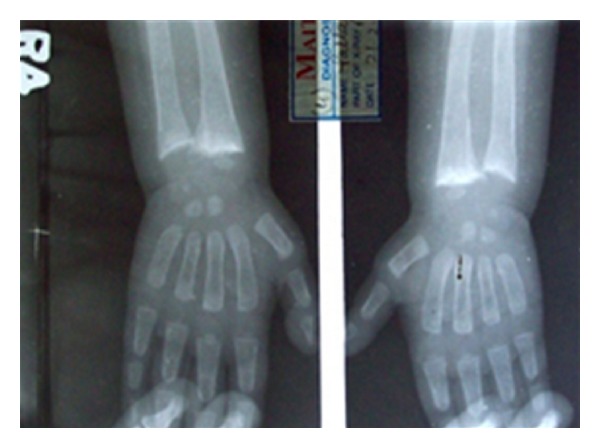
Showing wrist X-ray with features of active rickets.

**Figure 3:**
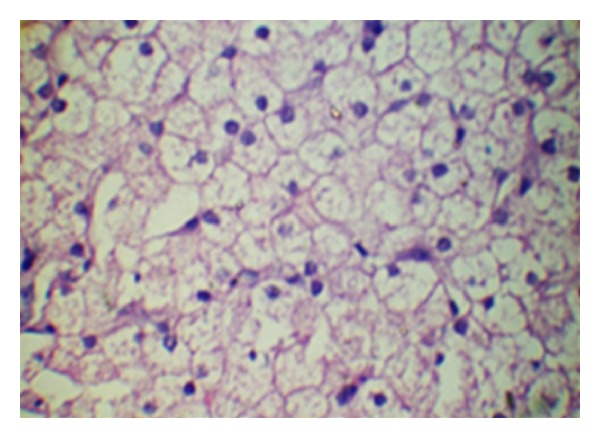
Showing histopathology of liver. Liver biopsy detected large pale staining hepatocytes with centrally placed nuclei giving appearance of plant cells and mosaic pattern with fibrous septa extending from portal tracts which as not entirely specific but suggestive of GSD.

Investigations revealed normal serum calcium (9.4 mg/dL), phosphorus was reduced to 2 mg/dL (normal 2.7–4.5 mg/dL), and markedly elevated serum alkaline phosphatase 1184 IU/L (normal 145–420 U/L) and normal potassium (4.1 meq/L). Fasting blood sugar was 80 mg/dl and two hours after eating, blood sugar was 96 mg/dL (normal 60–100). Thyroid profile was within normal limits. 25(OH) D level was 42 nmol/L (25–120 NMol/L) and 1, 25(OH)_2_D was within normal limit, it was 67 pmol/L (48–144 pmol/L). Parathormone level was mildly elevated; it was 6.2 pmol/L (normal 1.1–5.5 pmol/L). Fasting insulin level was 15 mcU/mL (normal values are 5–20 mcU/mL). Liver function tests were within normal limits. Serum cholesterol was 217 mg/dL (normal 45–182 mg/dL) and triglycerides were 268 mg/dL (normal 32–99 mg/dL) with normal serum HDL cholesterol and LDL cholesterol levels. Blood urea and serum creatinine, uric acid and lactate levels were within normal limits. Urine biochemistry showed pH of 6.2 (normal 4.5–8), specific gravity 1.026, glucosuria 2+, albumin 2+. Arterial blood gas detected; pH 7.22, HCO_3-_ 15, BE −6.7. Wrist X-ray showed features of active rickets (Figure 2). Ultrasonography of abdomen revealed hepatomegaly with normal echotexture; other organs were within normal limits. Liver biopsy was done which detected large pale staining hepatocytes with centrally placed nuclei giving appearance of plant cells and mosaic pattern with fibrous septa extending from portal tracts which not pathognomonic but suggestive of GSD (Figure 3).

 FBS was diagnosed and treatment started with supplementation of oral vitamin D_2_ (2000 IU/kg/24 hours as a single daily dose) and phosphate (0.6 g/24 hr) as Joulie's solution (4 mL given every 4 hourly, 5 times daily) for the hypophosphatemic rickets. Oral bicarbonate supplementation was also given as sodamint tablets (3 tablets three times a day) to correct the metabolic acidosis [2, 3]. Regarding diet, he was put on frequent and small meals with adequate caloric intake [2, 3].

**Figure 4:**
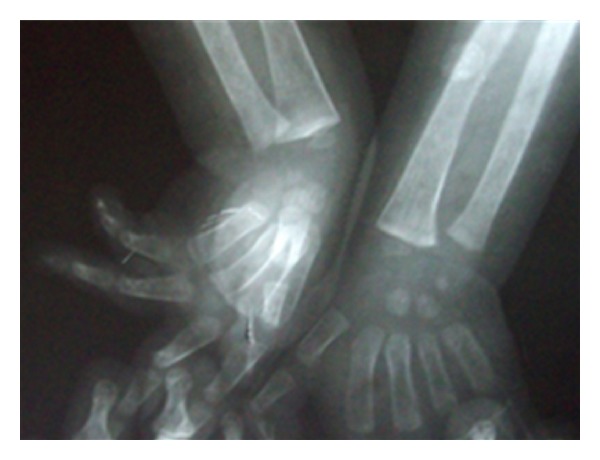
Showing wrist X-ray with features of healing rickets.

 Follow-up visit on 4 months; detected significant weight (3 kg) and gain in length (4 cm). Now he can stand with support. Repeated X-ray wrist showed significant recovery from rickets (Figure 4). However, it was not possible to assess histopathological improvement because parents did not give consent for repeat biopsy. Liver size did not change much on followup.

## 3. Discussion

FBS is a rare autosomal recessive disorder known since 1949 as hepatorenal glycogenesis with proximal renal tubular alteration [5–7]. 

Within the classification of GSD, FBS is denominated as type XI.

The common age of presentation is 2-month to 1 year. The index case was suffering from hypophosphatemic rickets which was the consequence of proximal renal tubular dysfunction. Other characteristic findings of proximal renal tubular dysfunction are glucosuria, phosphaturia, and bicarbonate loss and generalized aminoaciduria. That boy had huge hepatic enlargement which was due to hepatic accumulation of glycogen. Other clinical features of FBS are characteristic such as face (moon-shaped or doll-like face), growth retardation, and protuberant abdomen. Although, our patient was normoglycemic, fasting ketotic hypoglycemia and postprandial hyperglycemia are commonly noted due to low hepatic uptake [8, 9]. Hypoglycemia is frequently present in patients of FBS but chances of hypoglycemic seizure and mental retardation are less [10]. The pathogenesis is not definitively known; ketosis might have some neuro-protective role. Although the index case had normal fasting insulin level, other report has shown hypoinsulinemia due to altered sensitivity of *β* cells of pancreas to glucose. Parental consanguinity was absent in index case but it is observed in 2/3rd of patients of FBS. Due to economic constrain, genetic study was not possible. However, genetic research has indicated that FBS is a single gene disorder (OMIM 227810) caused by defects in the facilitative glucose transporter 2 (GLUT2 or SLC2A2) gene mapped on chromosome 3q26.1-26.3, that codes for the glucose transporter protein 2 expressed in hepatocytes, pancreatic beta-cells, enterocytes, and renal tubular cells [7, 9]. Some of FBS cases were identified through neonatal screening for galactosemia measuring blood galactose in Guthrie test cards.

 At present no specific treatment is available. Treatment of rickets is done with vitamin D in the form of 1, 25 dihydroxy vitamin D_3_. Citrate up to 15 mEq/kg/day is given every 4 hours to maintain bicarbonate more than 20 mEq/dL and along with oral phosphate solution administration. Corn starch was not added in our patient as he is euglycemic till date. Other report has shown advantage of corn starch, because corn starch provides glucose in slow release form and also which do not require diseased metabolic pathway. Although the exert association of galactosemia with FBS has not been established, it is preferred to avoid galactose from diet till all metabolic reports are available. Overall prognosis for survival to adulthood seems to be favorable; in addition to Fanconi and Bickel's original patient, at least two more patients have reached adulthood in stable condition.

In our case, clinical, radiological, and biochemical parameters all suggested hypophosphatemic rickets and GSD was detected by liver biopsy. Metabolic acidosis was only mild, but low serum calcium, phosphorus, bicarbonate, and high excretion suggested renal tubular dysfunction. Although hypoglycemia was not found in our patient, other cases report has documented hypoglycemia.
